# Resting-state fMRI in temporal lobe epilepsy patients with cognitive impairment: A protocol for systematic review and meta-analysis

**DOI:** 10.1097/MD.0000000000027249

**Published:** 2021-10-15

**Authors:** Yi-ming Sun, Yu-xuan Peng, Quan Wen, Yu Dai, Xin-ru Liu, Xue-ping Yang, Qing Ye

**Affiliations:** Chengdu Eighth People's Hospital, China.

**Keywords:** cognitive impairment, fMRI, meta-analysis, protocol, systematic review, temporal lobe epilepsy

## Abstract

**Background:**

Temporal lobe epilepsy is a group of neurological diseases caused by the repeated abnormal discharge of brain neurons. Patients with this disease are often accompanied with cognitive impairment. However, the pathogenesis of the cognitive impairment remains unclear. Resting state functional magnetic resonance imaging is a kind of magnetic resonance imaging method based on blood oxygen level dependence. This can reflect the spontaneous brain functional activity of a human brain in the resting state. In recent years, a number of researchers have used resting state functional magnetic resonance imaging to study the changes in resting spontaneous brain function in patients with temporal lobe epilepsy with cognitive impairment (TLE-CI). However, due to the differences in sample and methodology, the results of these studies were inconsistent. Therefore, the present study aimed to investigate the characteristics of the resting spontaneous brain function in patients with TLE-CI through a meta-analysis.

**Methods:**

A search was conducted on electronic databases, including PubMed, Cochrane Library, EMBASE, Web of Science, China National Knowledge Infrastructure, WANGFANG DATA and Chinese Biomedical Literature Database, and Baidu scholar Database, from the establishment of the database to April 20, 2021. Randomized controlled trials that employed amplitude of low-frequency fluctuations/regional homogeneity to investigate the changes in resting spontaneous brain function in patients with TLE-CI were selected. Anisotropic effect size version of signed differential mapping was applied to perform the data analysis.

**Results:**

The study summarized the changes in spontaneous brain function in patients with TLE-CI.

**Conclusion:**

The conclusion for the functional cerebral alterations based on the latest studies will be provided.

## 1 Introduction

Epilepsy is a group of neurological diseases caused by the repeated abnormal discharge of brain neurons. This is a prevalent neurological disorder that affects approximately 50 million people worldwide.^[1]^ However, temporal lobe epilepsy (TLE) is a common refractory type of epilepsy, which refers to seizures that originate from the temporal lobe, including the hippocampus, amygdala, parahippocampal gyrus, and lateral temporal neocortex. Although under the control of anti-epileptic drugs, this can still affect human brain function in a slow and long-term manner. Furthermore, cognitive impairment is the major concern for these patients, such as damaging memory, attention, language, and executive control function.^[2–4]^ But precise data on its incidence remains unclear.^[5]^ At present, a number of studies have investigated the factors that contribute to cognitive dysfunction. It has been considered that these cognitive deficits are caused by complicated reasons, including anti-epileptic drugs, recurrent seizures, seizure severity, and organic brain disorders of the temporal lobe, seizure-induced head trauma, age of onset of epilepsy, and long duration of epilepsy.^[2,6,7]^ However, the pathogenesis of the cognitive impairment remains unclear.

In patients with TLE, MRI studies have reported abnormal brain structures in hippocampus, entorhinal cortex,^[8]^ thalamus,^[9]^ and fornix.^[10]^ Depending on voxel-based morphometry, white matter reductions ipsilateral to the seizure focus were also found in the temporopolar, entorhinal, and perirhinal areas.^[11]^ Based on these studies, TLE originates unilaterally from the medial temporal lobe; they may be propagated from other regions which project to limbic areas.^[12,13]^

The changes of whole brain function caused by epilepsy have also become a research hotspot. At present, functional magnetic resonance imaging (fMRI) is being gradually applied in the diagnosis and research of epileptic brain function, providing neurophysiological and pathological evidence for the changes in brain function in epileptic patients. Among these, resting state fMRI has been extensively used to analyze the differences in activation characteristics of various brain regions in resting-state epilepsy patients and normal control groups through regional homogeneity (ReHo) and amplitude of low-frequency fluctuations (ALFF).^[14]^ Various studies have investigated the resting-state networks in temporal lobe epilepsy with cognitive impairment (TLE-CI) and TLE with the default mode network: activation of the left dorsolateral pre-frontal cortex in patients with verbal memory^[15]^; substantial decrease in activation in the neocortical, hippocampal, and parahippocampal regions in patients with 24-hour delayed verbal memory retrieval^[16]^; greater activation in the contralateral hippocampus in patients with worse memory performance.^[17]^ However, the sample variables were small, and the results were inconsistent. Therefore, it is necessary to conduct a systematic review of the literature that evaluated the changes in brain functional activities in patients with TLE-CI in fMRI.

## 2 Methods

### 2.1 Study registration

This protocol was registered with the International Platform of Registered Systematic Review and Meta-Analysis Protocols (INPLASY). The registration number is INPLASY202130092 (Doi: 10.37766/inplasy2021.3.0092). The protocol is according to Preferred Reporting Items for Systematic Reviews and Meta-Analyses (PRISMA-P) statement guidelines.^[18]^ The results of this meta-analysis will be published in a journal or conferences.

### 2.2 Inclusion criteria

#### 2.2.1 Types of participants

Adolescents and adult patients diagnosed with TLE (left or right) and cognitive impairment, according to any recognized diagnostic criteria, were included into the study. All participants were treated for their condition.

### 2.3 Types of study

#### 2.3.1 Inclusion criteria

Randomized controlled trials that compared the fMRI cerebral alterations in TLE of patients with cognitive impairment and healthy controls; studies that reported the whole-brain results in 3-dimensional coordinates (x, y, z) for changes in standard stereotactic space (Talairach or MNI); studies that used thresholds for significance corrected for multiple comparisons or uncorrected with spatial extent thresholds.

#### 2.3.2 Exclusion criteria

Studies that only reported findings in regions of interests; studies that used coordinates relative to the analysis that employed small volume corrections in pre-selected regions of interests.

**Types of interventions:** None.

### 2.4 Search methods for the identification of studies

#### 2.4.1 Search strategy

A systematic literature search was performed in the following electronic databases, from the establishment of database to April 20, 2021: PubMed, Cochrane Library, EMBASE, Web of Science, China National Knowledge Infrastructure, WANGFANG DATA and Chinese Biomedical Literature Database, and Baidu scholar Database. Merely studies in the English and Chinese language were included for the study.

The following search terms were used: “Epilepsy, Temporal Lobe”, “Cognitive Dysfunction”, “Neurocognitive”, “Deterioration^∗^”, “Disorder^∗^”, “fMRI”, “functional MRI”, “ReHo”, and “ALFF”. The sample for the search strategy for PubMed is summarized in Table 1.

**Table 1:** Search strategy for PubMed.

Number	Search terms
1	Epilepsy, Temporal Lobe
2	Cognitive Dysfunction
3	And 1–2
4	Uncinate
5	Temporal
6	Or 4–5
7	Epileps^∗^
8	And 3,6
9	Dysfunction^∗^
10	Impairment^∗^
11	Decline^∗^
12	Or 9-11
13	Cognitive
14	And 12-13
15	Neurocognitive^∗^
16	Disorder^∗^
17	And 15-16
18	Disorder^∗^
19	Mental^∗^
20	And 18-19
21	Or 14,17,20
22	And 8,21
23	And 3,22
24	fMRI
25	functional MRI
26	ALFF
27	ReHo
28	Or 24-27
29	And 23,28

#### 2.4.2 Selection of studies

**Figure 1:**
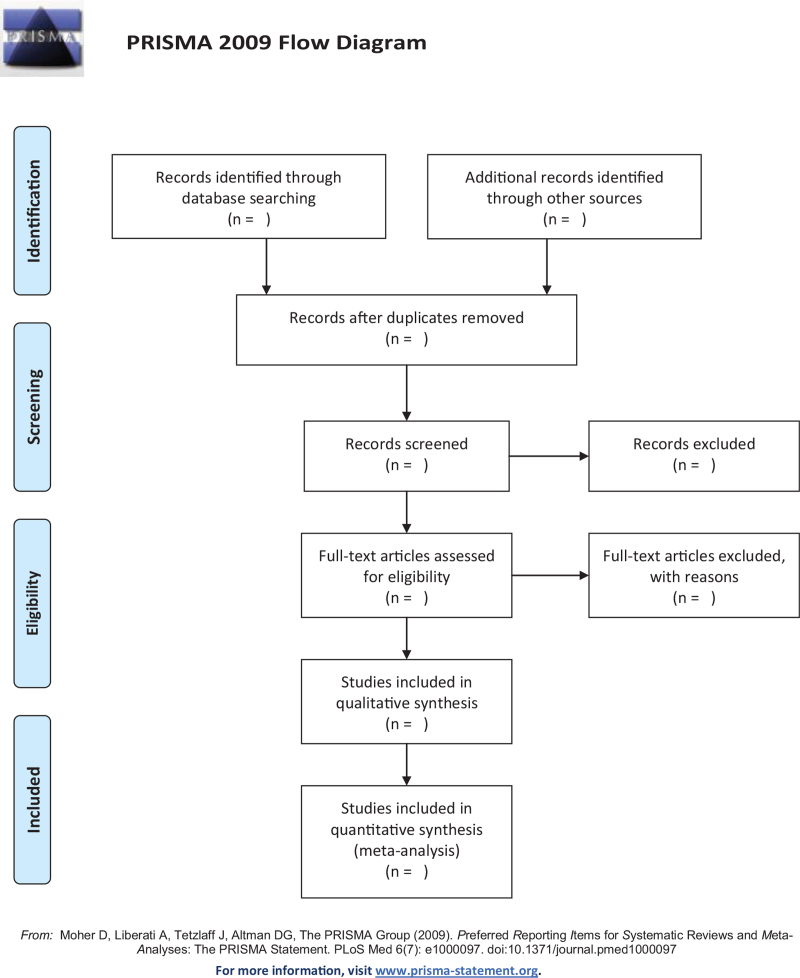
PRISMA flow chart.

The EndNote X9 document management software was used to filter the articles. Two researchers independently and carefully browsed the selected articles according to the inclusion and exclusion criteria. The reasons for the exclusion of articles was recorded, and any disagreements were resolved through the consensus of all authors. The diagram of the selection of studies is shown in Figure 1.

### 2.5 Data collection and analysis

#### 2.5.1 Data extraction and management

Two reviewers independently browsed the selected articles, and extracted and recorded the following information on Microsoft Excel: essential information: title, first author, publisher of the journal, year of publication, MRI type, and country; study design: inclusion and exclusion criteria, randomization method, the definition of cognitive impairment, statistical analysis technique and patient characteristics, and sample size; participants: gender, age, and disease duration; results and conclusion. At all stages, any disagreement was resolved by discussion with a senior author.

#### 2.5.2 Data synthesis and statistical analysis

The anisotropic effect size version of signed differential mapping (AES-SDM) software was used to analyze patients with TLE-CI and healthy controls through the ReHo or ALFFs between subjects. The peak coordinates in Talarach space were transformed into MNI space. In the experiment, if the result was a Z value, this was converted to the T value. AES-SDM was used to reconstruct the effect scales and statistical parameters for the increase and decrease in brain activation in each original study.

The Monte Carlo random number was set to 20. The full width at the half height of 20 mm was used to reconstruct the brain area difference map, and each voxel with a difference was given a corresponding weight value, which was as close as possible to the results of each study (if there were multiple assigned coordinates in a single study, the weighted value was adjusted). A meta-analysis was immediately performed after image reconstruction to calculate the voxel mass, with the statistical difference between TLE-CI patients and normal controls. In order to balance the sensitivity and specificity, the uncorrected *P* < .005 and voxel number ≥10 were used as the main thresholds.

### 2.6 Types of outcomes

Separate analyses on functional brain response abnormalities were conducted using AES-SDM. This method uses the peak coordinates to recreate a statistical parametric map for each study. Then, a map would be created using the effect sizes of the differences between patients and controls, and a standard random-effects variance-weighted meta-analysis was conducted for each voxel.

### 2.7 Assessment of risk of bias

The methodological quality of the randomized controlled trials was assessed using the risk of bias assessment tool, Newcastle-Ottawa scale. The total score of the scale was 9 points. If the score of the included literature was >6 points, this would be included into the study, while if the score was <6 points, it was excluded. This process was independently completed by 2 professional researchers. In case of any disputes, this was discussed by 2 researchers or resolved by a third researcher.

### 2.8 Sensitivity analysis

The Jack-knife sensitivity analysis method was used to confirm the repeatability of the results. That is, the statistical analysis was repeated, and a different dataset was removed each time. This tested the repeatability of the results.

### 2.9 Meta-regression or subgroup analysis

The meta-linear regression analysis method based on AES-SDM was used to explore the influence of age and disease duration in TLE-CI patients on the research results. The threshold range of *P* < .005 was set, and merely brain regions with a significant correlation between spontaneous brain function changes, and age and disease duration were reported. Finally, if adequate trials would be included, the following potential sources of heterogeneity would be explored using subgroup analyses or meta-regression: studies with low risk of bias, when compared to trials with high risk of bias; methods: ALFF\ReHo; scan T: 1.5\3.0.

### 2.10 Publication bias

If more than 10 studies were included, a funnel plot would be used to evaluate the publication bias. Egger or Begg test would be conducted to analyze the potential publication bias, and the results would be estimated based on the Cochrane Handbook for Systematic Reviews of Interventions.

### 2.11 Ethics and dissemination

No human or animal subjects or samples were/will be used. The results will be published in a peer-reviewed journal, and will be disseminated at local and international neurology conferences.

### 2.12 Patient and public involvement

Patients were not involved in the development of the research question, outcome measures, and study design.

## 3 Discussion

Nowadays, the mechanism of TLE cognitive impairment has not been fully elucidated, and studies suggest that cognitive impairment is closely related to the abnormal brain structure and function of patients. Resting state functional magnetic resonance imaging is used to observe the neural activities of various brain regions in the resting state, showing the interaction of brain neural networks. This paper aims to summarize the functional characteristics of the whole brain in patients with cognitive impairment of TLE, and to help us further understand the mechanism of cognitive impairment.

## Author contributions

**Conceptualization:** Qing Ye.

**Data curation:** Yiming Sun, Yuxuan Peng, Xueping Yang.

**Formal analysis:** Yu Dai.

**Funding acquisition:** Quan Wen.

**Investigation:** Xinru Liu, Qing Ye.

**Methodology:** Yuxuan Peng.

**Project administration:** Yiming Sun, Yu Dai.

**Software:** Yu Dai.

**Supervision:** Quan Wen.

**Validation:** Xinru Liu.

**Visualization:** Yiming Sun, Quan Wen.

**Writing – original draft:** Qing Ye.

**Writing – review & editing:** Yiming Sun, Qing Ye.
